# Prevalence and determinants of complementary and alternative medicine use among infertile patients in Lebanon: a cross sectional study

**DOI:** 10.1186/1472-6882-12-129

**Published:** 2012-08-20

**Authors:** Ghina S Ghazeeri, Johnny T Awwad, Mohamad Alameddine, Zeina MH Younes, Farah Naja

**Affiliations:** 1 Department of Obstetrics and Gynecology, American University of Beirut Medical Center Beirut, Beirut, Lebanon; 2 Department of Health Management and Policy, Faculty of Health Sciences, American University of Beirut, Beirut, Lebanon; 3 Department of Nutrition and Food Sciences, Faculty of Agricultural and Food Sciences, American University of Beirut, Beirut, Lebanon; 4 Department of Nutrition and Food Sciences Faculty of Agricultural and Food Sciences (FAFS), American University of Beirut, P.O.Box 11–0236, Riad El Solh, 11072020, Beirut, Lebanon

**Keywords:** Complementary and alternative medicine, Infertility, Lebanon

## Abstract

**Background:**

Complementary and alternative medicine (CAM) is widely used for the treatment of infertility. While the Middle East and North Africa region has been shown to house one of the fastest growing markets of CAM products in the world, research describing the use of CAM therapies among Middle-Eastern infertile patients is minimal. The aim of this study is to examine the prevalence, characteristics and determinants of CAM use among infertile patients in Lebanon.

**Methods:**

A cross sectional survey design was used to carry out face-to-face interviews with 213 consecutive patients attending the Assisted Reproductive Unit at a major academic medical center in Beirut. The questionnaire comprised three sections: socio-demographic and lifestyle characteristics, infertility-related aspects and information on CAM use. The main outcome measure was the use of CAM modalities for infertility treatment. Determinants of CAM use were assessed through the logistic regression method.

**Results:**

Overall, 41% of interviewed patients reported using a CAM modality at least once for their infertility. There was a differential by gender in the most commonly used CAM therapies; where males mostly used functional foods (e.g. honey & nuts) (82.9%) while females mostly relied on spiritual healing/prayer (56.5%). Factors associated with CAM use were higher household income (OR: 0.305, 95% CI: 0.132–0.703) and sex, with females using less CAM than males (OR: 0.12, 95% CI: 0.051–0.278). The older patients were diagnosed with infertility, the lower the odds of CAM use (p for trend <0.05). Almost half of the participants (48%) were advised on CAM use by their friends, and only 13% reported CAM use to their physician.

**Conclusions:**

The considerably high use of CAM modalities among Lebanese infertile patients, added to a poor CAM use disclosure to physicians, underscore the need to integrate CAM into the education and training of health professionals, as well as enhance infertile patients' awareness on safe use of CAM products.

## Background

Complementary and Alternative Medicine (CAM) refer to a heterogeneous group of practices that are defined by The National Center for Complementary and Alternative Medicines as “a group of diverse medical and health care systems, practices, and products that are not presently considered to be part of conventional medicine” [1]. According to the World Health Organization (WHO), more than three-quarters of the world’s population use complementary and alternative medicine (CAM) for health care. Up to date, there is still inadequate data concerning the potential benefits or risks attributed to the use of CAM [2]. Moreover, exchange of information on CAM use between the patient and physician has also been shown to be scarce [1]. The most reported reasons for the increase in the use of CAM include the absence of perceived efficacy and experience of adverse effects with conventional medicine, a perceived harmlessness of CAM treatments as well as the belief that use of CAM therapies allows users more active involvement in treatment and in healthcare decisions [3-5].

Infertility has been identified by the WHO as an international public health concern, affecting an estimated 72.4 million women in year 2007 [6,7].

While assisted reproductive technologies (ART) serve as promising treatment options for these patients to achieve parenthood, they do not always prove to be successful, and the expense associated with their utilization has proven to be a burden, leaving couples feeling “desperate” to try anything to conceive [4,5,8]. Previous studies about prevalence of CAM use among infertility patients reported estimates between 30 and 60% [1,9-11].

While the Middle East and North Africa region has been shown to house one of the fastest growing markets of CAM products in the world [12], research describing the use of CAM therapies among Middle-Eastern infertile patients is minimal. A recent study on the use of complementary and alternative medicines by a sample of Turkish women for fertility enhancement found that 82% of study participants have used CAM at least once for their infertility problem [4]. In Lebanon, no data exists on the prevalence, types, characteristics, and reasons for using CAM among infertile couples. With Lebanon showing the lowest fertility rate among the Arab countries [total fertility rate dropping from 3.2 in the year 2004 to 1.8 in the year 2009 [13-15], it may be hypothesized that couples are increasingly seeking alternative and complementary treatment modalities.

The lack of systematic information on the prevalence, types and mode of CAM use for the treatment of infertility in the Arab world in general, and Lebanon in particular, has hindered public efforts to enhance the safety of public consumption and slowed down the integration of CAM treatments into mainstream medicine.

The purpose of this study is to examine the frequency, types, modes and determinants of CAM use among couples seeking infertility treatment in Lebanon.

## Methods

A cross sectional survey was undertaken at the Assisted Reproductive Technology (ART) Unit at a major tertiary hospital in Beirut, American University of Beirut Medical Center, Lebanon. Ethical approval was obtained from the Institutional Review Board (IRB) at the American University of Beirut (AUB). “During the interview, a semi structured questionnaire was completed (Additional file 1)”. The ART unit, initiated in 1993, is the largest in the country, accepting patients rom fall of the Lebanese governorates and performing between 80 and 120 cycles per month, including intra-uterine insemination (IUI), in-vitro fertilization (IVF), and intracytoplasmic sperm injection (ICSI). The ART unit team consists of four specialized physicians, three embryologists and four nurses. Between January 2011 and August 2011, all patients (males and females) attending a routine visit at the ART unit were approached to complete an anonymous questionnaire about their use of CAM. When couples presented to the unit, the partner receiving treatment was invited to participate in the study. To be included in the study, patients had to be over 18 years of age, diagnosed with infertility for a minimum of one year and be conversant in either the English or Arabic language. All eligible patients were approached by a trained female nurse. Interviews took place in the visit room individually and lasted approximately 15 minutes. Subject’s written consent was obtained.

A face-to-face interview was undertaken with consecutive infertile patients attending the ART unit at AUB-MC. During the interview, a semi structured questionnaire was completed.

The first draft of the questionnaire was drafted by an expert panel including two physicians, a health management professor and an epidemiologist and was based on a synthesis of tools used in similar studies [4,10]. To enhance the content validity of this questionnaire and ensure its appropriateness to the local cultural context, a pilot study involving a sample of 15 subjects was carried out. Certain culturally sensitive items were identified and in turn were excluded, such as the number of sexual partners in the past and the age at which sexual activity was commenced. The final version of the questionnaire consisted of three sections. The first section included questions about the socio-demographic and lifestyle characteristics, such as age, sex, religion, number of years being married, education, income, living with parents-in-law, health insurance, smoking status (and number of years smoked) and self-perceived level of stress. Subjects were considered “insured” if they were found to have private and/or public insurance. Self-perceived stress was recorded as a number from 0 to 10, with a value of 0 being the lowest, indicating “no stress”, and a value of 10 being the highest, indicating “extreme stress” [16]. The second section of the questionnaire addressed aspects of the subject’s infertility, such as the age at diagnosis, kinship to spouse, presence of fertility problems in the family, duration of infertility, barriers to adherence to treatment, and conventional treatment modalities that have been utilized. In the last section of the questionnaire, participants were asked about the types of CAM treatments used since diagnosis with infertility for the purpose of improving their condition, frequency of use, the provider of CAM modalities, disclosure of CAM use to the physician, usefulness of CAM, side effects and purpose of use. For the purpose of this study, side effects referred to any unpleasant event that could be experienced by the patient as a result of his/her use of a specific CAM modality. This unpleasant event could range from psychological distress to serious medical condition. Use of CAM was defined as the use of any one or more CAM type for the purpose of infertility treatment since diagnosis.

The data was described by frequencies and percentages and by means and standard deviations for categorical and continuous variables respectively. The relationship between the various demographic and infertility-related characteristics and use of CAM was assessed by estimating odds ratios (OR) and 95% confidence intervals (CI) using logistic regression with “use of CAM” as the outcome variable. Multivariate logistic regression modeling was used to assess the predictors of CAM use. Variables were put in the model in order of strength of their association with CAM use as per the univariate analysis. The effect of each variable on the model was assessed and this variable was kept in if it significantly contributed to a better fit of the model. Statistical significance was set at p <0.05, with all tests being two-sided. SPSS 18.0 software was utilized to analyze the obtained data.

## Results

### Descriptive analyses

Out of the 262 patients approached, 213 agreed to participate in the study (response rate: 81.3%). Lack of sufficient time to spend on the interview and fear of name disclosure were the main reasons that were reported for the refusal to participate. Analysis of findings showed that 41% of patients have used a form of CAM at least once as a treatment for their infertility. When “prayer” was excluded from the definition of CAM, prevalence of CAM use in the study population became 23%.

Demographic, lifestyle, reproductive and infertility related characteristics of the study population are presented in Table 1. Study participants’ mean age was 34.7 ± 6.4 years, with no significant difference between users and non users of CAM. Among the socio-demographic factors considered in this study, sex, years of marriage, income, education and religion were found to be significantly associated with the use of CAM. The odds of using CAM were significantly lower among females (OR: 0.24, CI: 0.13–0.44). Being married from more than 7 years was associated with a significantly higher odd of using CAM (OR: 2.35, CI: 1.21–4.56). Patients with monthly income ranging between $1000 and $2000 and greater than $2000 were less likely to use CAM as compared to patients with income less than $1000. Higher education (university and college levels) was associated with lower prevalence of CAM use (OR: 0.35, CI: 0.20–0.62). Use of CAM was significantly lower among Christian participants as compared to Muslim patients (OR: 0.44, CI: 0.21–0.92). As for infertility related characteristics, prevalence of CAM use decreased with increasing age at diagnosis with infertility and increased with longer duration of infertility (OR: 1.11, CI: 1.04–1.18).

**Table 1:** Demographic, lifestyle, reproductive and infertility-related characteristics of users and non-users of CAM treatments*

	**Overall (n = 213)**	**Users of CAM (n = 87)**	**Non-users of CAM (n = 126)**	OR (95% CI)**
**Age (years) (**mean ± SD)	34.7 ± 6.4	34.9 ± 6.7	34.6 ± 6.3	1.01 (0.97-1.05)
**Sex** (n,%)				
Male	63 (29.6%)	41 (47.1%)	22 (17.5%)	1
Female	150 (70.4%)	46 (52.9%)	104 (82.5%)	0.24 (0.13-0.44)
**Religion** (n,%)				
Muslim (Sunni, Shiite, Druze)	164 (78.5%)	74 (86.0%)	90 (73.2%)	1
Christian	45 (21.5%)	12 (14.0%)	33 (26.8%)	0.44 (0.21-0.92)
**Years Married** (n,%)				
1-3 years	97 (45.5%)	34 (39.1%)	63 (50.0%)	1
4-6 years	57 (26.8%)	20 (23.0%)	37 (29.4%)	1.00 (0.51-1.99)
≥ 7 years	59 (27.7%)	33 (37.9%)	26 (20.6%)	2.35 (1.21-4.56)
*p for trend*				<0.001
**Household Income** (n,%)				
<$1000/month	90 (42.3%)	51 (58.6%)	39 (31.0%)	1
$1000-2000/month	47 (22.1%)	17 (19.5%)	30 (23.8%)	0.43 (0.21-0.90)
>$2000/month	76 (35.7%)	19 (21.8%)	57 (45.2%)	0.26 (0.13-0.50)
*p for trend*				<0.001
**Educational level** (n,%)				
Illiterate to Baccalaureate	86 (40.4%)	48 (55.2%)	38 (30.2%)	1
Diploma/University	127 (59.6%)	39 (44.8%)	88 (69.8%)	0.35 (0.20-0.62)
**Living with parents-in-law** (n,%)				
No	196 (92.0%)	77 (88.5%)	119 (94.4%)	1
Yes	17 (8.0%)	10 (11.5%)	7 (5.6%)	2.21 (0.81-6.05)
**Health Insurance** (n,%)				
Uninsured	59 (27.7%)	29 (33.3%)	30 (23.8%)	1
Insured	154 (72.3%)	58 (66.7%)	96 (76.2%)	0.63 (0.34-1.15)
**Smoking status** (n,%)				
Non-smoker^**a**^	177 (83.1%)	68 (78.2%)	109 (86.5%)	1
Smoker	36 (16.9%)	19 (21.8%)	17 (13.5%)	1.79 (0.87-3.68)
No. of years (mean ± SD)	16.0 ± 7.4	17.2 ± 7.3	14.6 ± 7.4	1.05 (0.96-1.16)
**Self-perceived stress**^**b**^				
≤5	103 (48.4%)	39 (44.8%)	64 (50.8%)	1
>5	110 (51.6%)	48 (55.2%)	62 (49.2%)	1.27 (0.73-2.20)
**Age at diagnosis** (mean ± SD)				
< 20 years	19 (8.9%)	14 (16.1%)	5 (4.0%)	1
20-30 years	91 (42.7%)	38 (43.7%)	53 (42.1%)	0.26 (0.09-0.77)
>30 years	103 (48.4%)	35 (40.2%)	68 (54.0%)	0.18 (0.06-0.55)
*p for trend*				<0.001
**Kinship to spouse** (n,%)				
Not related	183 (85.9%)	72 (82.8%)	111 (88.1%)	1
Related	30 (14.1%)	15 (17.2%)	15 (11.9%)	1.54 (0.71-3.35)
**Family fertility problems** (n,%)				
None	174 (81.7%)	72 (82.8%)	102 (81.0%)	1
Yes	39 (18.3%)	15 (17.2%)	24 (19.0%)	0.89 (0.43-1.81)
**Duration of Infertility** (mean ± SD)	4.3 ± 4.8	5.6 ± 5.7	3.4 ± 3.9	1.11 (1.04-1.18)
**Barriers to adherence to treatment**				
No barriers	85 (39.9%)	32 (36.8%)	53 (42.1%)	1
Barriers	128 (60.1%)	55 (63.2%)	73 (57.9%)	1.25 (0.71-2.19)
**Current treatment (Males & Females)** (n,%)				
No IUI or IVF treatment^**c**^	43 (20.2%)	14 (16.1%)	29 (23.0%)	1
IUI or IVF procedure	170 (79.8%)	73 (83.9%)	97 (77.0%)	1.56 (0.77-3.16)

*Values in this table represent n and % for the categorical variables and means ± SDs for the continuous variables.

** OR and their 95% CI were derived using a univariate logistic model with CAM use as the dependent variable.

^**a**^ Indicates both non-smokers and past-smokers.

^b^ Self-perceived stress was assessed using a scaling system ranging from 0–10 (0 = no stress at all, 10 = extreme stress).

^c^ Includes patients not receiving any treatment, patients receiving medications without IUI and/or surgery.

The types and modes of CAM used by study participants are presented in Table 2. Types of CAM were divided into dietary supplements and spiritual/prayer therapy. Dietary supplements included vitamins and minerals, over the counter supplements, herbal therapies and functional foods. Included among the over the counter supplements are fish oils such as salmon and cod liver oils, omega-3 supplements, and products from general nutrition centers. Herbal therapies include plants that are consumed for their claimed health benefits, and encompass remedies such as feverfew, garlic, black seed, ginger, ginkgo, and Asian ginseng.[17] “Functional foods” included any foods that were perceived as having a fertility-enhancing property beyond their basic function; such as honey, and foods containing essential fats (e.g. fish, nuts and seeds). For the purpose of this study, spiritual/prayer therapy referred to any religion-related activity practiced to improve fertility. The most commonly used CAM treatment among males was found to be functional foods (82.9%), whereas spiritual therapy or “prayers” and herbal therapies, were the most commonly used CAM treatments among females (56.5% and 43.5% for spiritual and herbal therapies, respectively).

Among the CAM users, 90.2% of the males reported using CAM in order to improve their sperm characteristics (sperm motility, production and/or quality) and 87% of female participants reported using CAM in order to enhance their chances of achieving a pregnancy. Other reasons for CAM use reported by patients included relaxation, enhancing psychological well-being or strengthening energy.

When asked about how they chose the CAM modality used, almost half of the participants indicated referral by friends (48.3%) and influence by media (33.3%). Only 6.9% (n = 6) of patients used CAM because of a referral by a health care practitioner. The use of CAM among study participants was regular for the majority of patients (two or more times per week for a minimum of one month) (70.1%). Factors that drove patients to utilize CAM were “belief in its advantages” (56.3%), followed by “trying CAM because of a suggestion” (51.7%), with a few reporting their uptake of CAM to be due to “dissatisfaction with conventional medicine” (9.2%) and feeling that they “had no other alternative” (11.5%). Out of the 87 users of CAM, only 11 patients (12.6%) reported their use of CAM to their physician. Reported reasons for not using CAM were that the physician had not prescribed them (53.2%), followed by the lack of belief in the benefits of CAM as enhancers of fertility (48.4%) (Table 2).

**Table 2:** Types and modes of CAM use among couples seeking infertility treatment (n = 87)

	**Frequency n (%)**
Types of CAM modalities used among males (n,%)*	
Functional foods	34 (82.9%)
Spiritual/ prayer therapy	13 (31.7%)
Vitamins and minerals	12 (29.3%)
Herbal therapies	5 (12.2%)
Over the counter supplements	2 (4.9%)
Types of CAM modalities used among females (n,%)*	
Spiritual/ prayer therapy	26 (56.5%)
Herbal therapies	20 (43.5%)
Over the counter supplements	16 (34.8%)
Vitamins and minerals	7 (15.2%)
Reasons for CAM use among males (n,%)*	
Improvement of sperm characteristics	37 (90%)
Relax, feel better psychologically and provide energy	12 (29.3%)
Relief of other reproductive related conditions	2 (4.9%)
Reasons for CAM use among females (n,%)*	6 (6.9%)
Enhance chances of pregnancy	40 (87%)
Relax, feel better psychologically and provide energy	19 (41.3%)
Uterine and ovulatory related reasons	17 (37%)
Relief of other reproductive related conditions	3 (4.3%)
How CAM was chosen (n,%)*	
Personal choice	22 (25.3%)
Friends	42 (48.3%)
Media (Internet, magazines, TV)	29 (33.3%)
Health practitioner	6 (6.9%)
Health food shop	2 (2.3%)
Family beliefs	28 (32.2%)
**Frequency of CAM use (n,%)**	
Only once	12 (13.8%)
Regularly (2 or more per week for min a month)	61 (70.1%)
Rarely	14 (16.1%)
Why have you used CAM (n,%)*	
Disappointment from conventional medicine	8 (9.2%)
Feeling of having no alternative	10 (11.5%)
Belief in the advantages of CAM	49 (56.3%)
Trying because of a suggestion	45 (51.7%)
**Feeling after CAM use (n,%)**	
Feeling stronger, physically better and in good psychological condition	43 (49.4%)
No change or physically worse and in bad psychological condition	44 (50.6%)
**Usefulness of CAM (n,%)**	
Not at all	32 (36.8%)
Some	29 (33.3%)
A lot	15 (17.2%)
Can’t tell	11 (12.6%)
**Reporting CAM use to the doctor (n,%)**	
No	76 (87.4%)
Yes	11 (12.6%)
**Side effects from CAM use (n,%)**	
No	81 (93.1%)
Yes	6 (6.9%)
**Use of CAM again**	
No	34 (39.1%)
Yes	53 (60.9%)
**Recommend CAM to other infertile patients**	
No	42 (48.3%)
Yes	45 (51.7%)
Reasons for not using CAM among non-users (n = 126) *	
Never heard of it	6 (4.8%)
Afraid of the side effects	25 (19.8%)
Do not believe in it	61 (48.4%)
The doctor didn’t prescribe it	67 (53.2%)
Not to have additional burden	12 (9.5%)
Other**	26 (20.6%)

*Columns do not sum to 100% due to the option of multiple answers.

**Includes: waste of time, lack of proof of their benefit, not in need for them yet, lack of advice on their use, knowing the problem is purely medical, presence of co morbidities, unsafe, unhelpful in this case, prefer conventional treatment.

Results of the multivariate logistic regression analysis examining the association of the various demographic and infertility-related characteristics and CAM use are presented in Table 3. Sex, age at diagnosis with infertility, household income, and type of infertility treatment were independent predictors of CAM use. Prevalence of CAM use was lower among females (OR: 0.12, CI: 0.051–0.278). In addition, the older the patients were diagnosed with infertility the less likely they were to use a CAM modality (p for trend < 0.001). Subjects belonging to the highest income bracket (>2000$/month) used less CAM therapies as compared to those in the lowest income bracket (<1000$/month) (OR: 0.305, CI: 0.132–0.703). Subjects undergoing IUI or IVF procedures were more likely to be CAM users (OR: 20.918; CI: 1.1–7.75). Age, barriers to medical treatment adherence, years of marriage, and religion were not found to reach statistical significance after multivariate adjustment.

**Table 3:** Multivariate Logistic Regression Model with OR estimates and 95% CI for the association between demographic and fertility-related factors and the use of CAM among study participants (n = 213)

	**CAM use**	
	**OR**	**95% CI**
**Age**	1.00	0.93-1.09
**Sex**		
Male	1	---
Female	0.12	0.05-0.28
**Age at Diagnosis**		
< 20 years	1	---
20-30 years	0.17	0.05-0.60
> 30 years	0.09	0.02-0.39
*p for trend*	<0.001	
**Barriers to adherence to treatment**		
No barriers	1	---
Barriers	0.96	0.48-1.95
**Religion**		
Muslim (Sunni, Shiite, Druze)	1	---
Christian	1.06	0.42-2.65
**Years Married**		
1–3 years	1	---
4-6 years	0.92	0.40-2.11
≥7 years	2.08	0.91-4.78
*p for trend*	<0.001	
**Household Income**		
<1000 $	1	---
1000-2000$	0.43	0.18-1.04
>2000$	0.31	0.13-0.70
*p for trend*	<0.001	
**Type of treatment**		
No IUI or IVF treatment	1	---
IUI or IVF procedure	2.92	1.10-7.75

## Discussion

This study investigated the prevalence and determinants of CAM use among infertile patients attending the Assisted Reproductive Technology (ART) Unit at the American University of Beirut Medical Center, a major tertiary hospital in Beirut, Lebanon. We found that 41% of infertile patients used a form of CAM at least once as a treatment for their infertility. Studies on prevalence of CAM use among infertile patients reported a wide range of estimates. Similar to our findings, a study from the United Kingdom assessing CAM use among 400 infertile women found 40% of participants to have used CAM as a therapy for their failure to conceive [11]. Furthermore, in Denmark, a prospective observational cohort study reported a 31% prevalence of CAM use among infertile women [1]. However, in the United States, a lower prevalence was found in a cohort study of 428 couples seeking infertility treatment (29%) [10], while a relatively high prevalence (82%) of CAM use among Turkish infertile patients was recently reported [4]. Possible reasons for the discrepancy in prevalence estimates reported in the literature could be the heterogeneity of CAM practices, where there may be differences in what is defined as a CAM treatment and what types of CAM modalities are included in the studies. Other reasons may be differences in study design as well as in cultural backgrounds which could lead to differential patterns of use.

Our findings showed that among women, “spiritual healing”, specifically “prayer” and “religious vows”, were the most commonly practiced CAM therapies. Religious vows involve making a promise to perform a specific deed once the prayer has been answered. Lebanon seems to be a country whereby religion is highly influential on people’s daily practices, with prayer being an integral part of the culture. Spiritual healing has been suggested to have potentially beneficial effects through its provision of hope and in turn, a positive attitude towards patients’ conventional infertility treatment, as was suggested in the Turkish study assessing CAM use for infertility enhancement [4].

In this study, the most commonly utilized CAM therapy by men was “functional foods”, specifically fish, nuts, and seeds, honey and royal honey. Seeds and nuts have been reported by complementary medicine practitioners to be beneficial for treating infertility due to their polyunsaturated fatty acid content [18]. Honey has been traditionally used among the Lebanese and other Arab populations for treatment of many diseases, such as the common cold and cough, in addition to infertility. Royal honey, also called “food for queens”, is a honey bee secreted from the glands in the hypopharynx of worker bees that is believed to give the queen bees their longevity and fertility. Furthermore, honey is mentioned in both the *Quran* (the Muslim Holy Book) and the Holy Bible as having the feature of healing mankind [19,20].

In addition to prayers and functional foods, our results showed that herbal therapies were used by men and women for infertility treatment, including blackseed, ginseng, maca, and marjoram. Blackseed, the common name for *Nigella sativa* L. seeds, is traditionally used as a spice and food preservative. Although hexane extracts of this seed have been shown to have significant antifertility activity in rats [21], it is a common belief among muslims and Christians in Lebanon and other countries of the Middle East that this seed possesses curative abilities for a wide range of diseases, including fertility [22]. Ginseng, maca and marjoram are herbal plants that have been suggested in the literature to confer beneficial effects on male fertility [23-25].

Although minimal or no risk has been associated with prayer and the use of functional foods for infertility treatment, herbal therapies have been reported to adversely affect chances of pregnancy and health. Phytoestrogens present in herbal supplements have been suggested to have negative estrogenic effects on implantation [1]. Furthermore, the side effects associated with a number of herbs is still unknown [2].

It is disconcerting that only 7% of the CAM users in our sample reported being advised on CAM modalities by their “health practitioner”, and the disclosure rate of CAM use to the physician was found to be low (13%). These findings are consistent with previous research, where in a review of qualitative and quantitative studies evaluating the disclosure of patients’ CAM use to medical practitioners, Robinson & McGrail [26] reported disclosure rate to be as low as 23% in some of their included studies. Possible reasons for why patients seem to find it difficult to report their CAM use to their physician include the fear of receiving a negative reaction and disapproval from their physician, believing that their physician did not need to know about their CAM use, and/or because their physician had not inquired about their CAM use [26,27]. This low disclosure rate of CAM use is alarming and may warrant the need to train physicians on probing their patients on CAM use [22]. Health care practitioners need to become more aware of the increasing prevalence of their patients’ CAM use in order to be able to improve the provision of evidence-based knowledge to their patients concerning the use of these therapies [28].

Our results indicated that sex, age at infertility diagnosis, household income and the type of infertility treatment were independent correlates of CAM use. Males were found to be at higher odds of using CAM. This is in contrast to other studies that found women to be at higher odds of using CAM compared to men [11,27,29]. A possible explanation of our finding could be the fact that in the Arab culture, including Lebanon, fertility is linked to manhood, leading male infertility to be an issue for masculinity, marriage and family life [30]. As such, males in this culture may be placing more attention on treatments to enhance their fertility as a mean to restore their “manhood” [30]; such assumption would need to be substantiated in future sociological/ anthropological research.

Our results showed that higher income was associated with lower use of CAM therapies. In previous studies of determinants of CAM use, household income was consistently positively correlated with CAM use [10,11,31]. This relationship between CAM use and a high income status in other studies could be attributed to the fact that couples of high income would be more likely to afford the cost of CAM, in addition to their infertility treatments [11]. In those studies, the most widely used CAM were costly modalities, such as acupuncture and massage therapy. In our study population on the other hand, the most widely used CAM modalities were functional foods and prayers among male and female patients, respectively; both of which are therapies that are considered of lower cost, and thus may be less likely to pose as a financial burden to patients of lower income.

Several limitations are to be considered in this study. First, the fact that recruitment of participants took place in an Assisted Reproductive Technologies Unit might have led to an underestimation of the prevalence of CAM use since surveyed subjects have a potential bias toward conventional treatment. Second, given that participants in this study were recruited from one clinical setting raises questions concerning the generalizability of our findings; however, the ART unit at the American University of Beirut Medical Center is considered the largest unit for infertility treatment in Lebanon (as reflected by the high number of cycles it performs per month) and is a major referral center for the treatment of infertility from all other governorates. Third, the cross sectional nature of the study does not allow establishing causality between the various correlates and the CAM use. Finally, the possibility of a recall bias cannot be ruled out in self reports concerning CAM use.

## Conclusions

Our results showed that a considerable proportion of patients used CAM therapies for infertility treatment. “Functional foods” and “spiritual healing” were the most commonly used CAM modalities by our sample of male and female patients, respectively. CAM use is associated with lower household income, male gender, younger age at infertility diagnosis, and undergoing ART procedures for infertility treatment. The considerably high use of CAM modalities among Lebanese infertile patients, added to a poor CAM use disclosure to physicians suggests the need for physicians to initiate discussions with their patients regarding CAM use. In addition future research is needed in areas such as CAM education of healthcare workers and patients’ awareness on the proper use of the CAM modalities.

## Competing interests

The authors declare that they have no competing interests.

## Authors’ contribution

GG assisted in the design of the study and acquisition of data, in addition to writing the first draft of the manuscript. FN conceived of the study, participated in the design of the study, evaluated the results and corrected the manuscript for publication. JA helped in drafting the manuscript and discussion of results. MA conducted the statistical analysis. ZY contributed in obtaining the completed questionnaire and assisted to analyzing the data. All authors read and approved the final manuscript.

## Pre-publication history

The pre-publication history for this paper can be accessed here:

http://www.biomedcentral.com/1472-6882/12/129/prepub

## Supplementary Material

Additional file 1 Questionnaire used to assess the prevalence and determinants of the use of complementary and alternative medicine (CAM) among Lebanese infertile patients. Click here for file
