# Supplementary material for: Prevalence and determinants of complementary and alternative medicine use among infertile patients in Lebanon: a cross sectional study
Source: BMC Complement Altern Med. 2012 Aug 20;12:129. doi: 10.1186/1472-6882-12-129 (PMC3512516; doi:10.1186/1472-6882-12-129)
Supplement: Additional file 1 — Questionnaire used to assess the prevalence and determinants of the use of complementary and alternative medicine (CAM) among Lebanese infertile patients. [file 1472-6882-12-129-S1.docx]

|  |
| --- |

**Prevalence and determinants of the use of complementary and alternative medicine (CAM) among Lebanese infertile patients**

**Questionnaire**

CAM is defined as prevention, diagnosis, treatment, and healthcare practices which are not an integral part of the conventional medicine. It complements mainstream medicine by contributing to a common whole, by satisfying a demand not met by orthodoxy or by diversifying the conceptual frame works of medicine.

| **1. Date/time (dd/mm/yy) ___/___/___** |
| --- |

**Subject ID: _____________________**

**SECTION A: DEMOGRAPHICS**

| **____________:Age .1** | | |
| --- | --- | --- |
| **2. Birth place** | | |
| City .c | Town .b | Village. a |

| **:Place of residence .3** | | |
| --- | --- | --- |
| Other .b | Beirut .a | |
| **:Sex .4** | | |
| Female .b | | Male .a |

| **:Religion .5** | | | | | | | |
| --- | --- | --- | --- | --- | --- | --- | --- |
| d. Refuse to answer | c. Other | b. Muslim | | | a. Christian: | | |
|  |  | iii. Other, please specify ……… | ii.Shi’ite | i. Sunnite | iii. Other, please specify: ……… | ii. Orthodox | i. Catholic |

| 1. **6. Years married:** | | | | |
| --- | --- | --- | --- | --- |
| d. > 10 years | c. 7-10 years | b. 4-6 years | | a. 1-3 year |
| 1. **7. Annual household income:** | | | | |
| b. 500- 1000$ per month | | | a. <500 $ per month | |
| d. >2000$ per month | | | c. 1000-2000 $ per month | |

| 1. **8. Highest education level attained:** | | |
| --- | --- | --- |
| e. Masters, Doctoral | c. Secondary, Baccalaureate | a. Illiterate |
|  | d. Diploma; University: Bachelor | b. School: Primary |

| **: 9. Employment status** | | |
| --- | --- | --- |
| e. Unemployed | b. Self-Employed | a. Employed (Employee) |
|  | d. Housewife | c. Retired |

| 1. **10. Living with parents-in-law:** | |
| --- | --- |
| b. No | a. Yes |

| 1. **11. Number of bedrooms in the household: _____________** 2. **12. Number of persons in the house: _______________** | | |
| --- | --- | --- |
| 1. **13. Health expenses coverage:** | | |
| c. Uninsured | b. Insurance; Private | a. Insurance; Public |

| 1. **14. Intake of coffee/day:** | | | |
| --- | --- | --- | --- |
| d. > 2 cups/day | c. 1-2 cups/day | b. <1 cup/day | a. No intake |

| 1. **15. Intake of soft drinks:** | | | |
| --- | --- | --- | --- |
| d. > 2 cups/day | c. 1-2 cups/day | b. <1 cup/day | a. No intake |

| **16. Smoking status (cigarettes):** | | | |
| --- | --- | --- | --- |
| d. Current smoker (>1 pack/day) | c. Current smoker (< 1 pack/day) | b. Past smoker | a.Non-smoker |

| **17. Smoking status (Arguileh):** | | |
| --- | --- | --- |
| e. > Once/day | c. 2-3 times/week | a.Non-smoker |
|  | d. Once/day | b.≤ Once per week/Occasionally |

| **18. If you are a smoker of cigarettes or arguileh, please indicate the number of years you have been a smoker:** ……… |
| --- |
| **19. On a scale of 0 to 10, where 0 means no stress at all and 10 means extreme stress, please indicate your self-perceived stress level:** |
| **10 9 8 7 6 5 4 3 2 1 0** |

| **SECTION B: Infertility** |
| --- |
| **20. Age at diagnosis of infertility: ________________** |

| **21. Kinship to wife/husband:** | | | | | | | | | | | | | | | | | | |
| --- | --- | --- | --- | --- | --- | --- | --- | --- | --- | --- | --- | --- | --- | --- | --- | --- | --- | --- |
| b. Closely related | | | | | | | | a. Not closely related | | | | | | | | | | |
| **22. Relationship between parents and/or grandparents:** | | | | | | | | | | | | | | | | | | |
| c. Both parents and grandparents are related | | | | b. Parents or grandparents are related | | | | | | | | | | | | a. None are related | | |
| **23. Are there any fertility problems in your immediate family?** | | | | | | | | | | | | | | | | | | |
| b. None | | | | | a. Yes | | | | | | | | | | | | | |
| **24. Duration of infertility (in years):** | | | | | | | | | | | | | | | | | | |
| **25. Diagnosis of infertility:** | | | | | | | | | | | | | | | | | | |
| c. Male and female factors | | | | | 1. Female factor only | | | | | | | | | | | | | |
| d. No male or female factor identified | | | | | 1. Male factor only | | | | | | | | | | | | | |
| **26. Total number of pregnancies (whether successful or not):** | | | | | | | | | | | | | | | | | | |
| c. More than once | | | b. Pregnant for the first time or have been pregnant once | | | | | | | | | | | | | | a. Never | |
| **27. Number of births:** | | | | | | | | | | | | | | | | | | |
| e. Given birth  ≥ 5 times | | c. Currently in the first pregnancy (> 20 weeks) | | | | | a. None | | | | | | | | | | | |
|  |  | d. Given birth ≥ 2 times | | | | | b. Never completed pregnancy beyond 20 weeks | | | | | | | | | | | |
| **28. Do you suffer from any other health condition?** | | | | | | | | | | | | | | | | | | |
| c. Cancer chronic | | | | | b. Cardiovascular disease | | | | | | | | | a. Hypertension | | | | |
| f. Other, please specify: ………… | | | | | e. Diabetes | | | | | | | | | d. Obstructive pulmonary disease | | | | |
| **29. Have you ever been treated for your infertility?** | | | | | | | | | | | | | | | | | | |
| b. No | | | | | | | | | a. Yes | | | | | | | | | |
| **30. If yes, what was the type of treatment (for women)?** | | | | | | | | | | | | | | | | | | |
| e. Surgery | c. Intrauterine insemination only | | | | | | | | | a. Medications for ovulation induction | | | | | | | | |
|  | d. In vitro fertilization | | | | | | | | | b. Medications without intrauterine insemination | | | | | | | | |
| **31. If yes, what was the type of treatment (for men)?** | | | | | | | | | | | | | | | | | | |
| e. Intrauterine insemination | | | | c. Hormonal therapy | | | | | | | | | | a. Lifestyle modification | | | | |
| f. In vitro fertilization | | | | d. Micro-surgical correction (Vasovasostomy, Vasoepididymostomy, Vericocelectomy) | | | | | | | | | | b. Medications  (alpha-adrenergic agonists) | | | | |
| **32. If you were treated for your infertility, please indicate the number of treatment cycles:** ………….. | | | | | | | | | | | | | | | | | | |
| **33. Do you adhere to your doctor’s recommendations?** | | | | | | | | | | | | | | | | | | |
| c. Sometimes | | | | b. No | | | | | | | a. Yes | | | | | | | |
| **34. What are the main barriers to your adherence to the recommendations?** | | | | | | | | | | | | | | | | | | |
| d. Others, please specify………….. | | | | c. Intolerance of drug side effects | | | b. Inconvenient scheduling of the medication | | | | | | | | | a. Unaffordable medication | | |
| **35. How do you describe your overall physical health?** | | | | | | | | | | | | | | | | | | |
| c. Good | | | | | b. Fair | | | | | | | | a. Poor | | | | | |
| **36. What in your opinion is the cause of your infertility?** | | | | | | | | | | | | | | | | | | |
| e. Other, specify: ……….. | | | | | | d. Heredity | | | | | | c. Weight | | | b. Lifestyle | | | a. Age |

| **SECTION C: CAM USE** | | | | | | | | | | | | | | | | | |
| --- | --- | --- | --- | --- | --- | --- | --- | --- | --- | --- | --- | --- | --- | --- | --- | --- | --- |
| **37. Have you used CAM since your diagnosis of infertility?** | | | | | | | | | | | | | | | | | |
| b. No | | | | | | | | | a. Yes | | | | | | | | |
| **38. Have you used CAM in the previous year?** | | | | | | | | | | | | | | | | | |
| b. No | | | | | | | | | a. Yes | | | | | | | | |
| **39. If you have not used CAM, would you consider using it in the future?** | | | | | | | | | | | | | | | | | |
| b. No | | | | | | | | | a. Yes | | | | | | | | |
| **40. If you have not used CAM, why not?** | | | | | | | | | | | | | | | | | |
| e. Not to have additional burden | | | | c. I don’t believe in it | | | | | | | | | | | | | a. I never heard of it |
| f. Other, please specify:………. | | | | d. The doctor didn’t prescribe it | | | | | | | | | | | | | b. I’m afraid of the side effects |
| **41. Have you asked your doctor about the CAM product you used?** | | | | | | | | | | | | | | | | | |
| b. No | | | | | | | | | a. Yes | | | | | | | | |
| **42. How did you choose your CAM?** | | | | | | | | | | | | | | | | | |
| c. Media (Internet, magazines, TV) | | | | | | b. Friends | | | | | | | | a. Personal choice | | | |
| f. Health food shop | | | | | | e. Family beliefs | | | | | | | | d. Health practitioner | | | |
| **43. How often do you use CAM?** | | | | | | | | | | | | | | | | | |
| c. Once per month | | | | | | a. One time | | | | | | | | | | | |
| d. Other | | | | | | b. Regularly (2 or more per week for a minimum of a month) | | | | | | | | | | | |
| **44. Who provided you with the CAM treatment?** | | | | | | | | | | | | | | | | | |
| e. Homeopath | c. Practitioner of traditional medicine | | | | | | | | | | a. Massage therapist | | | | | | |
|  | d. Naturopath | | | | | | | | | | b. Acupuncturist | | | | | | |
| **45. If the use of CAM was regular, how much do you estimate the cost is per month?** .................... | | | | | | | | | | | | | | | | | |
| **46. Why have you used CAM?** | | | | | | | | | | | | | | | | | |
| c. Belief in advantages of complementary and alternative medicine practices | | | | | | | b. Feeling of having no alternative | | | | | | a. Disappointment from conventional medical therapy | | | | |
| e. Other, please specify:……………… | | | | | | | | | | | | d. Trying because of a suggestion | | | | | |
| **47. What was your feeling after you have used CAM:** | | | | | | | | | | | | | | | | | |
| d. Feeling physically worse | | | c. Feeling of disappearance of several symptoms | | | | | | | b. Feeling of being in good psychological condition | | | | | | a.Feeling of strengthening of body | |
| h. Other, please specify  ........................ | | | g. Feeling of no change | | | | | | | f. Feeling rise of several symptoms | | | | | | e. Feeling of being in bad psychological condition | |
| **48. In general, how much did the treatment help you? Would you say:** | | | | | | | | | | | | | | | | | |
| d. Can’t tell | | c. A lot, very satisfied | | | | | | b. Some | | | | | | | a.Not at all | | |
| **49. Have you suffered from any side effect?** | | | | | | | | | | | | | | | | | |
| c. Undecided | | | | | b. No | | | | | | | | | a. Yes | | | |
| **50. Would you use CAM again?** | | | | | | | | | | | | | | | | | |
| c. Undecided | | | | | b. No | | | | | | | | | a. Yes | | | |
| **51. Will you recommend the use of this CAM to other infertility patients?** | | | | | | | | | | | | | | | | | |
| c. Undecided | | | | | b. No | | | | | | | | | a. Yes | | | |

****For women, please proceed to answer questions 52 – 53***

****For men, please proceed to answer questions 54 - 55***

| **52. What type of CAM product have you used?** | | | | |
| --- | --- | --- | --- | --- |
| c. Herbal remedies/Herbal preparations, specify:  .............................. | | b. Dietary supplements or (Special foods) | | a.Vitamins/Minerals |
| f. Other, please specify………….: | | e. Folk medicine | | d. Spiritual healing |
| **53. Was the use of CAM for:** | | | | |
| 1. Help me relax and feel better psychologically | | 1. To enhance my chances of a pregnancy | | |
| d. Getting relieved from sorcery spell | | c. Increase production of eggs | | |
| f. Help my ovaries release a monthly egg  (stimulate egg release) | | e. Improve my uterus’s ability to accept and harvest the fertilized egg | | |
| i. Other, please specify…………………… | h. Providing energy | | g. Make me feel more in control of my treatment and more actively involved | |

****For men:***

| **54. What type of CAM product have you used?** | | |
| --- | --- | --- |
| e.Honey | 1. Minerals/ Vitamins (including antioxidants such as   vitamin C & vitamin E) | |
| f. Spiritual healing | 1. Dietary supplements or Special foods   (including carnitine supplements) | |
| 1. Folk medicine | 1. Foods containing essential fats such as fish, nuts, and/or seeds | |
| h.Other, please specify………….: | 1. Herbal remedies/Herbal preparations, specify: | |
| **55. Was the use of CAM for:** | | |
| 1. Improve sperm count and/or motility | | 1. Induce/increase sperm production |
| 1. Decrease the production of sperm toxins (fight sperm antibodies) | | |
| e. Getting relieved from sorcery spell | | d. Improve sperm quality |
| g. Help me relax and feel better psychologically | | f. Providing energy |
| 1. Other, please specify…………………… | | 1. Make me feel more in control of my treatment and more actively involved |

**THANK YOU for your cooperation and time**
